# Physiological Psychology

**Published:** 1857-04-01

**Authors:** Robert Dunn


### Art. VIII.—PHYSIOLOGICAL PSYCHOLOGY.

### No. IV.

BY ROBERT DUNN, F.R.C.S. ENG.

*Continued from No. V., p. 156.*

*Intellectual Consciousness.*—"A scientific psychology," says Waitz, "should exhibit the laws according to which the life of the human mind is evolved; that is, it should point out the common basis upon which *all mental life* rests, follow the threads by means of which all its phenomena are connected with each other, show the germs out of which they spring, and how they unfold themselves, into that multiplicity and richness of *inner life* which are manifested in the mature man."*

We have passed in review some of the leading phenomena of the sensational and perceptive consciousness, and attempted to specialize the nervous apparatus through which they are respectively manifested. We have seen that ideation is the first stage in our intellectual progress, and we have glanced at its general bearings, in relation to our composite nature, as animal, moral,

* *Vide* Waitz's Prospectus to his "Lehrbuck," quoted by Morrell in his "Elements of Psychology."

and intellectual beings. But knowledge that is *definite*, *exact*, and *communicable* belongs to a still higher phase of mental development than that of intuitive feeling and perceptive experience,—of the sensational and perceptive consciousness, which we have hitherto been considering. For in world-consciousness, as in self-consciousness, there is an individuality, an inward or subjective experience which is *unutterable* and *incommunicable.* The primary intuitions of all our perceptive faculties, even in regard to the phenomena of nature, but still more especially in respect to our social and moral relations, are closely interwoven with feeling, and, indeed, are often intensely felt, but on this very account they are *incommunicable;* for they cannot be *articulately expressed*, nor adequately conveyed by any system of signs from one mind to another. We can only judge of the intuitive feelings and perceptive experience of others by what we ourselves experience. No words can convey their equivalents; that is, can make others feel a sensation which we feel, or experience an inward light which reveals to us the primary elements of knowledge. There is, indeed, for the expression of absolutely individual feelings and emotions, a universal language, common to man and the lower animals—the language of Nature—*of inarticulate cries and of gesticulation.* The interjection comes nearest to this, but it has a certain amount of generality about it. In the case of the unfortunate deaf-mutes, the paramount importance of gestural language we see strikingly exemplified. To them, in their state of isolation and normal condition, left without the peculiar instruction which their situation requires, it is everything; independent of all conventional arrangements, and addressing itself principally, if not solely, to the sight, it is their only mode of communication with others. Thus, for instance, when they have beheld the raging passion of anger in another, and seen the swollen features, the distorted visage, the convulsed limbs; in a word, all the violence of action *visible* in anger, they can only tell of this to others by imitating the contortions and reacting the scene which they have witnessed. There cannot, indeed, be a doubt that "our minds are subject to a variety of feelings, and that the effects of these are visible in the features, attitudes, and gestures. Every distinct emotion has its appropriate expression, and thus a language *altogether independent of words exists*, displayed by the countenances and actions of man. Every person is aware of the bodily expression of fear, love, joy; and one can seldom ever mistake or confound the language of those with that of courage, hatred, or sorrow. Such language is immediately and instinctively recognised in every state of civilization, from the American savage to the most refined citizen. The haughty step, the erect carriage, and disdainful look, are always sure indications of pride; in the timid gait and sidelong look fear is at once perceived; while agony is always too fearfully portrayed in the distorted looks and agonized features of severe suffering. This language addresses itself to the sight; the deaf and dumb therefore are able to avail themselves perfectly of its use; and thus it possesses for them, through life, always a charm which written language appears rarely to acquire."*

But knowledge, to be definite, exact, and communicable to others, must pass through the process of abstraction, and become embodied in the forms and symbols of the understanding, in fine, *in spoken or written language.* The intuitions of our perceptive faculties, *our idealized impressions*, which have been stored up in the memory as *mental images*, and *reproduced as representative ideas,* after having been associated, and when sufficiently generalized, have again to be projected out of the mind, to be externalized, and by the imaginative faculty, ideality, to be embodied in objective realities. But when once symbolized, or embodied in signs, our generalized ideas are no longer mere subjective representations; for, being thrown into fixed and significant types, which perform, though imperfectly, the office of abstract ideas, they exist in the mind, altogether apart from the region of immediate and inward experience, *as independent intellectual realities;* and, as such, become distinct and intelligible objects of contemplation, which can be placed at pleasure, either within or without the consciousness of the moment. In perception, as we have seen, ideation is effected in response to impressions made upon us *from without*, by virtue of the primeval harmony which exists between our perceptive faculties and external nature; but here *the mental process is reversed*, for the mind, separating itself from outward restraints, and impelled by its own inherent intellectual activity, by ideality, it embodies its inward images and representative ideas in objective realities. And this *objectifying* of our *inward* or *mental ideas* is all important to our progress in knowledge. For, "until signs are employed, our mental images are not held clearly apart; they merge, like dissolving views, into one another. Our life, in fact, without them, would be more like a dream than a waking reality—portions of a thousand different ideas perpetually combining with and melting into one another. Language, on the other hand, forms a new world, in which all our mental processes are *objectified*, held clearly apart, and not only made distinct to our-

* "The Deaf and Dumb; their Position in Society, and the Principles of their Education Considered." By W. P. Scott, M.D., Principal of the West-of-England Institution for the Education of the Deaf and Dumb. London. 1844.

selves, but so embodied as to be rendered likewise separate intellectual realities to other minds as well."*

When once, however, our intellectual activity, instead of *objectifying* our *inward images* in *existing outward* realities, constructs for itself the *sign, phonetic or visible*, for the embodiment of the *intellectual idea*—"*the sign for the thing signified*"—the mind reaches a still higher phase of development. For in the construction and through the instrumentality of language the mind rises above feeling, above perception, above all the inward images of the imagination, and creating a new external world of its own, into which it transfers the phenomena of its inner life, it achieves the first step in the *freedom of human thought*. "In language, the sign, whether spoken or written, is *objective*; it appeals to the *senses*; it comes to us from the outward world, and is constructed from the elements of nature around us. At the same time, it has no natural meaning, and contains no thoughts apart from the mind which created or uses it. Its whole essence consists in its being the embodiment of an idea; in brief, *it is idea objectified*."†

Language is thus an intellectual instrument intermediate between perception and thought, and written notional words are the symbols or representatives of objectified ideas. All notional words, indeed, belong to the region of representative ideas, after these ideas have attained their most general character; and though words cannot excite the feelings like a gesture, nor warm the imagination like a picture, they are the indispensable machinery in the process of generalization and abstraction. Through them we grasp the essential elements which distinguish one thing from another, and classify our multifarious experiences. "In this way it is that they serve to construct the more general outline of knowledge. Hence the wonderful power which words possess on the whole process of thought; hence the capacity they attain, after the teachings of experience have paved the way, for expressing the very essence of the things to which they relate; hence, too, their use in forming a broad platform, on which the results of all the lower processes of mind are plainly recorded, and from which we can commence those higher forms of activity, which give to reason its all but infinite range and all but omnipotent force."‡

To the unfortunate, but educated deaf mutes, cut off from "hearing the mirror of speech," and denied the gratifications which flow from the interchange of ideas through the medium of "sweet sounds," written language is *speech in visible forms*, and written words, as the symbols or representatives of *objectified ideas*, are regarded by them as units, in the same way as we

* Morrell's "Psychology," p. 184. † Ibid. ‡ Ibid.

regard letters, the various objects around them being so many simple objects of thought. "In the minds of the deaf mutes, written words," says Dègenèrado, "awaken the conception of things themselves, in the same manner as they awaken in ours the conception of sounds, with this difference, that polysyllabic words recal to them but a single idea, while to us they record a number of sounds at once." Nor can there exist a doubt that our alphabetic writing, losing its *phonetic* character, becomes to them truly *ideographic.**

Their association, however, of ideas with written language must necessarily give to it a very different character from that which obtains with us, who enjoy the blessings of speech and hearing. So universal, indeed, is the practice of associating *ideas with sound,* it is not without difficulty that we can conceive the possibility of associating written characters with ideas without the intervention of sound. We learn to speak long before we learn to read or write; and thus, in the natural order of things, articulate sounds become the representatives of ideas, and written characters the representatives or symbols of sounds. Besides, hearing and sound are fitted to each other, and are in such intimate relationship, that hearing has been aptly designated "the mirror of speech." And thus it is, that while in articulate speech the mental image or intellectual idea, which has been moulded for expression in the organ of language, finds utterance by the lips, through the agency of the volitional power, the articulate sound—the spoken word—is reflected back, and returns again by *hearing* through the ears, first to the perceptive, and thence to intellectual consciousness.

The function of articulate speech is the exclusive prerogative of man, and language is common to all the races of man. It is the crowning gift of his beneficent Creator; "for to be without language, spoken or written, is almost to be without thought. We must not think, in a speculative comparison of this sort, of mere savage life; for the rudest savages would be as much superior to a race of beings without speech, as the most civilized nations at this moment are, compared with the half-brutal wanderers of forests and deserts, whose ferocious ignorance seems to know little more than how to destroy or to be destroyed. In our social intercourse, language constitutes the chief delight —giving happiness to hours, the wearying heaviness of which must otherwise have rendered existence an insupportable burden. In its more important character, as fixed in the imperishable

* Jerome Condon, a learned professor of Pavia, so early as the sixteenth century, says,—"Writing is associated with speech, and speech with thought; but written characters and ideas may be connected together *without the intervention of sounds,* as in hieroglyphic characters.—See "Journal of Education," No. 6, p. 204.

records which are transmitted in uninterrupted progression from that generation which passes away to the generation which succeeds, it gives to the individual man the product of all the creative energies of mankind, extending even to the humblest intellect, which can still mix itself with the illustrious dead, the privilege which has been poetically allotted to the immortality of genius, of being 'the citizen of every country, and the contemporary of every age.' "*

It is as natural for man, constituted as he is, and endowed with the faculty of speech, when vividly affected, to give expression, and to find utterance in articulate sounds, to his feelings, emotions, ideas, and thoughts, as it is for him voluntarily to use his locomotive powers in progression. But the scream of alarm, the shriek of horror, and the laugh of surprise, like the scowl of hatred, are *natural signs*, and *not conventional ones, like articulate words.* Still, thought and language are almost inseparably associated, and it has been well observed—"Were a family of men to be created by a miracle in a wilderness, they would, if similarly endowed like ourselves, *feel the impulse of the faculty of speech*, and soon learn, in the first instance, to comprehend each other's gestures and cries, and other signs of natural language, and ascend by these means to the exalted acquisition of an artificial language, by giving, step after step, conventional names to objects and actions, emotions and passions, generalizations and abstractions."† Thus, to the natural language of inarticulate sounds, gestures, and actions, would be added the conventional language of signs, until, in the fulness of time, alphabetical writing and the invention of printing *consummated* the benefits derived from the *noble prerogative of speech.*

Gall was the first to enunciate that the cerebral seat of the *faculty of speech* is in the anterior lobes of the brain ; and since his time, as I have elsewhere observed,‡ a great mass of evidence has been collected in support of his localization of the organ.

"In 1848, two memoirs were read before the Académie Nationale de Médecine de Paris—one by M. Belhomme, 'De la Localisation de la Parole dans les Lobes Antérieurs du Cerveau ;' and the other, by M. Bouillaud, entitled 'Nouvelles Récherches Cliniques Propres à Demontrer que le sens du Langage Articulé et le Principe co-ordinateur des Mouvements de la Parole resident dans les Lobules Anté-

* Brown's Lectures on the "Philosophy of the Mind."

† "Memoir of Dr. Spurzheim." By A. Carmichael, M.R.S.A.

‡ Case of Hemiplegia, with cerebral softening, and in which loss of speech was a prominent symptom. By Robert Dunn, F.R.C.S. Read before the Royal Medical and Chirurgical Society of London, June 25, 1850, and published in the "Lancet," Oct 22 and Nov. 2, 1850.

rieurs du Cerveau,' containing new observations made by him since the date of his former paper, in 1839.

"The subject has undergone much discussion in France, and opposing evidence has been adduced. Andral* gives the particulars of two cases—one, in which loss of speech was the only cephalic symptom; and another, where it was complicated with hemiplegia of the right side, but the intellect was unaffected. They were both in old women, the first eighty, and the other seventy-three years of age. In the first case the speech was lost all at once, but not in a fit, three years before her death. She was never known to have lost her consciousness, nor the power of sensation or motion. Andral says—'Tout semblait nous annoncer que l'intelligence avait son intégrité. Dans les quatre membres, les mouvemens étaient libres, faciles, et la malade sentait bien les impressions douleureuses qu'on cherchait à faire naître sur la peau qui les recouvre. Lorsqu'on lui demandait si elle souffrait de la tête, ou si elle en avait souffert, elle repondait par un geste negatif. L'ouïe, la vue, et l'odorat, s'accomplissaient comme dans l'état normal.' At the autopsy, in the left hemisphere there was found a small ramollissement, of the size of a large pea—'Au niveau et en dehors de l'extremité postérieure du corps strié tout-a-fait à sa pointe;' and in the right hemisphere a similar ramollissement—'A l'union de la moitié antérieure avec la moitié postérieure de cet hémisphère, à une égale distance de ces bords interne et externe, et au point de jonction des deux tiers supérieurs avec le tiers inférieur de la masse nerveuse située du centre ovale de Vieussens.' These were the only cerebral lesions. In the second case—'Dans tout l'encephale, il n'y a d'altéré que le corps strié du côté gauche.' It was a soft, pulpy mass to within three lines of its exterior surface. Andral observes—'Le siége du ramollissement est digne de remarque; il est exactement borné à l'un des corps striés, ce qui n'empêche pas qu'il n'y ait paralysie des deux membres et abolition de la faculté de parler.' Other cases have been recorded, in which the structural lesion was confined to the corpora striata, and a few in which the middle and posterior lobes were implicated in the disease of the striated bodies.

"But, in the consideration of this subject, it is never to be forgotten that the perfect power of speech—that is, the power of giving utterance to our thoughts and ideas in suitable and appropriate language, *depends upon the due relation between the centres of intellectual action, and of the encephalic motor centres, through which the volitional power is exercised.* Thoughts or ideas may be moulded for expression in the seat of intellectual action, but the due agency of the volitional power, to give them utterance, requires the integrity of the commissural fibres, and of the motor centres, through which the volitional impulses of thought operate in speech. The imperfect power of articulation which we so constantly meet with in hemiplegic patients, I have no doubt is owing to some structural lesion in the integrity of the motor centre of volition; and hence does it not necessarily follow that loss of speech or power of utterance will alike result from *disease of the*

* "Clinique Médicale—Maladies de l'Encephale, Vol. v. p. 454.

*anterior lobes, or of such parts of the corpora striata as are in direct relation with them?*

"There is not, I believe, a single instance on record in which the power of utterance was retained *intact*, however sound and healthy the great hemispherical ganglia may have been found, where the corpora striata were both diseased. The apparently conflicting evidence which has been adduced as to the seat of the faculty of speech admits of a satisfactory explanation, when thus considered in relation to the centres of intellectual action and the motor centres of volition."

I brought this view of the subject under the notice of the Royal Medical and Chirurgical Society in a paper, "On a Case of Hemiplegia with cerebral softening, and in which the loss of speech was a prominent symptom," read June 25, 1850,* and I may here reiterate, my own mind rests in the conviction, that the amount of pathological and other evidence which has been amassed, irresistibly establishes the position of Gall, as to the site of the organ of the faculty of language in the anterior lobes of the brain, and that the power of articulate speech, that is, of giving utterance in appropriate language to our thoughts, feelings,

* The case was that of a lady advanced in years, who had suffered from three attacks of apoplexy. The first occurred in October, 1844, seemed "congestive" in its character, and passed away without any other permanent consequences than this, that she continually used one word for another, not applying appropriate names to the things or persons she desired to signify. The second attack, in May, 1847, left her permanently hemiplegic on the right side, the power of voluntary motion being completely abolished, and but little sensibility being preserved, though reflex movements could be excited, in the lower extremity, by tickling the sole of the foot. For the remainder of her life she remained altogether *incapable of speech*, not being able to say *yes* or *no* in reply to a simple question, and never getting beyond the utterance of the monosyllable *dat—dat;* yet all her senses were intact; *the motions of the tongue were free, and there was no difficulty of deglutition.* She did not seem to have lost any of her intellectual powers; but her emotional sensibility was rather increased. Her general health continued good up to the time of the last fatal seizure, which occurred in April, 1850, without any premonitory symptoms.

At the *post-mortem* examination, the upper two-thirds of the *anterior lobe* of the *left hemisphere* was found to be in a state of complete destruction, with colourless softening; while the middle and posterior lobes were sound and healthy. The *right hemisphere* was healthy; but the greatest change was in the ganglionic masses, at their base, and in the commissural structure. The upper half of the corpus striatum on the *left side* was destroyed by softening; the optic thalamus was shrunken to less than half its natural size, its upper surface being greatly wasted; while, on the *right side*, a small and recent apoplectic clot was seen on the upper and anterior surface of the corpus striatum, the whole of the upper half of which was in a state of *ramollissement;* while on the outer surface of the thalamus also were noticed some indications of white softening. The corpus callosum was destroyed, except at its anterior and inferior reflexion, and the anterior commissure and fornix were gone. Microscopic examination of the softened parts presented an abundance of compound cells and of fatty matter in the capillaries. In this case it is quite evident that, with the disorganization of the left anterior lobe, its functional power was entirely abolished; and though the right hemisphere was healthy, and there is every evidence, from the history of the case, that it maintained and exercised its functional power as a centre of intellectual action, still the volitional agency was *wanting to give* utterance to the passing thought, for the corpus striatum on the same side was not in its integrity.

and emotions, requires the integrity of the *corpora striata, and their commissural fibres, as the motor channels, through which the will or volitional power operates in speech.* A striking and instructive illustration was presented, in the young woman's case to which I have so frequently alluded, of the dependance of the power of utterance in articulate speech upon the due relation between the centres of intellectual action and of the motor centres, through which the will operates in speech. In her case, the perfect integrity of the corpora striata was abundantly manifest, for they were in the full play of their functional power, as *motor centres*, but she was *speechless* so long as the perceptive and intellectual faculties were in abeyance. Ideas, indeed, are the pabula of thought, and articulate speech is the interpreter and minister of thought. As we are now constituted, our thoughts are invariably clothed and find utterance in speech; but without ideation, without mental images and representative ideas, there could be no thoughts; and *without thought language would cease.* But thought there may be, and in the case of the unfortunate and uninstructed deaf mutes, thought there is, independent of, and without language. Nay, without speech, man, by virtue of his perceptive organs, and intellectual faculties, can observe objects, and mentally arrange, associate, and form them into groups. He can judge of their properties and qualities,—compare them, and even deduce inferences;—but how weak and incomplete are these processes of thought when language is wanting! Without language the mighty triumphs which science has achieved over nature would have been impossible; and without the machinery of words, how limited and contracted would be the process of generalization and abstraction! Language implies a train of thinking. We reproduce in speech the mutual relations of objects, the relations of our thoughts to objects, and, the order and relation of our thoughts themselves. Words, as we have seen, are purely conventional; they have no natural meaning of their own, and contain no thoughts apart from the the mind, which created and uses them.

They are, in fact, the final expressions of that mental process as well as the depository of its final results, consummated through the instrumentality of the faculty of language, by which knowledge becomes *definite*, *exact*, and *communicable*, and through which the mind, elevated above the region of mere ideation, increases in intellectual activity and rises to a higher phase of development—that of *thought* and *reason*. Logic expounds the *laws of thought* and *the art of reasoning*. But, as the *instrument of thought and reasoning*, the value and importance of language cannot be overrated. For language implies a train of thinking;—it is the circulating medium of our thoughts—

the minister of thought and its interpreter. Words are the materials of thought. For our mental images, *reproduced* in the memory as representative ideas or conceptions, when embodied in the conventional symbols of words, become *fixed and definite objects of thought,* and such they are to *all* who use them. Nay, according to Leibnitz, words are sometimes more than the signs or symbols of thought—*they become thoughts.* Such are his *symbolical cognitions or conceptions.* Among all the races of man, the instinctive impulse is irresistible to give utterance in articulate sounds to his feelings, emotions, and thoughts; and not only to fix upon articulate sounds, or names, as the representatives of his *intuitive cognitions* or *conceptions of things,* but also to find expressions for *the different qualities and states of things.* From such beginnings, "to all the uses and powers of articulate sounds and artificial language, how exalted is the ascent! how immense the efficacy and enjoyment possessed by man! the intercommunion of minds in social or scientific converse—the force and perspicacity of argument advanced to such a degree by general terms and intellectual abstractions—the strains of poetry, inculcating piety, magnanimity, and virtue—the thunders of eloquence, commanding the destinies of nations, and involving in its splendid career the interests both of time and eternity."*

The constructive faculty of language in continuous speech, involving, as it does, *the power of combining words together,* so as to express the mutual relations of objects—the relations of our thoughts to objects, and the order and relation of our thoughts themselves, thus enables us, through our reasoning and reflecting faculties, to judge explicitly of these relations, and to frame a method by which our judgments may be articulately expressed. And in this way it is that continuous speech becomes moulded, step by step, into a complete organ of thought, and that "a sentence or proposition in language answers to a complete thought in psychology. By a *complete thought,* in the sphere of the understanding, is meant, a *distinct act of comparison between two terms, in which we apprehend the relationship that exists between them.* All logical or formal thought answers to this explanation; and *the mental activity,* by which we compare terms, find out their exact agreement or disagreement, give expression to this in propositions, and deduce other popositions from them, is that which, *par excellence,* bears the title of The Understanding."†

* Carmichael, *ante cit.* † Morrell's "Psychology."

(*To be continued*).

